# A new alien mantis in Italy: is the Indochina mantis *Hierodula patellifera* chasing the train for Europe?

**DOI:** 10.3897/BDJ.8.e50779

**Published:** 2020-03-04

**Authors:** Roberto Battiston, Rachele Amerini, William Di Pietro, Luis Alessandro Guariento, Luca Bolognin, Enzo Moretto

**Affiliations:** 1 Musei del Canal di Brenta, Valbrenta, Italy Musei del Canal di Brenta Valbrenta Italy; 2 Department of Physical Geography and Ecosystem Science, Lund University, Sölvegatan, Sweden Department of Physical Geography and Ecosystem Science, Lund University Sölvegatan Sweden; 3 Associazione Culturale Arthropoda Live Museum, Sesto San Giovanni, Italy Associazione Culturale Arthropoda Live Museum Sesto San Giovanni Italy; 4 Department of Biology, University of Padova, Padua, Italy Department of Biology, University of Padova Padua Italy; 5 5 Esapolis Invertebrate Museum & Butterfly Arc, Padua, Italy 5 Esapolis Invertebrate Museum & Butterfly Arc Padua Italy

**Keywords:** aliens species, distribution pattern, railways, population dynamic, identification, new records, biological invasion

## Abstract

The presence of the Indochina mantis *Hierodula patellifera* (Mantidae, Mantinae) as a new alien species in Italy is reported, with the description of the first stable macro-population in Europe. This macro-population shows a wide distribution, comprising several fragmented and reproducing sub-populations in Northern Italy and one in Southern France. Specimens and individuals were collected or observed on trees and ornamentals in urban ecosystems with the help of citizen science. A spatial analysis (Average Nearest Neighbour) was undertaken to characterise the present distribution pattern, evidencing the hot spots of arrival and the local spreading process. The random pattern of presence in the local urban textures and the resistance of this species to the challenging North Italian climate, are here discussed in the perspective of a future expansion to central and Northern Europe, using probably the main railways to arrive at depots and cities, travelling with Asian goods. Identification characters are also presented to separate this alien species from the other species of the subfamily Mantinae, native or introduced, present in Europe.

## Introduction

During the last few years, some mantids species have drawn the attention of specialists and the media in Europe as new alien species. This role, even if not common, is not new for this order of insects at a global level (Gurney 1951, Anderson 2018, Howarth and Mull 1992, Ramsay 1984), but has been encountered in Europe as a fast evolving scenario (Marabuto 2014, Battiston et al. 2017, Battiston et al. 2018, Battiston et al. 2019, Schwarz and Ehrmann 2018, Moulin 2020). Some of the new European aliens have arrived from other countries or even continents (e.g. *Brunneria borealis* Scudder, 1896, *Miomantis caffra* Saussure, 1871, *M. paykullii* Stål, 1871 and *Tenodera sinensis* Saussure, 1871), but the causes of their arrival are not clear (Schwarz and Ehrmann 2018). Some others have more intricate dynamics like *Sphodromantis viridis vischeri* (Werner, 1933) and *Hierodula tenuidentata* Saussure, 1869, pertinent to an arrival from neighbouring areas which was caused or facilitated probably by human activities (Battiston et al. 2018, Battiston et al. 2019, Cianferoni et al. 2018). In this new scenario, some Euro-Mediterranean native species, like *Ameles spallanzania* (Rossi, 1792) and *Mantis religiosa* Linnaeus, 1758), are expanding their natural distribution from the Southern regions to the North, probably due to contribution from human-mediated transport (Battiston and Buzzetti 2012).

Regarding the genus *Hierodula*, while the presence of *H. tenuidentata* in Italy is now well established in the Po valley and still spreading, the distribution patterns of a second species of *Hierodula*, the Indochina mantis *H. patellifera* Serville 1839, remains to be verified. This species was described from Java (Indonesia) and it is distributed in China, India, Japan, Korea, Nepal and several Pacific islands (Ehrmann and Borer 2015). It has been recently recorded in Northern Italy on a few individuals collected in 2018 (Battiston et al. 2019) and its presence as a new alien was supposed but not evaluated. Indeed, the record of the few individuals could be accidental (e.g. due to an occasional/unconventional trade from Asia or the release/escape from pet shops or breeding) and the presence of a stable and vital population was not reported. Recently, the species has been documented as a new alien species in Europe on some specimens collected in a restricted area in Southern France (Moulin 2020). Here new and abundant records from different localities of Northern and Central Italy and different years are presented, which define the presence of vital populations and include this species as a new alien of the mantidofauna of Italy and Europe.

## Material and methods

New presence records and, when possible, individuals of *H. patellifera* have been collected from 2015 to 2019, mostly using citizen-science. Citizens have been involved using fact-sheets on the alien mantid species present in Italy and materials and records have been posted and discussed on the main social networks (Facebook and Instagram), following the observations and interacting with the users, helping them with the identification and tracking the records. Field inspections, done often together with the observers, have been undertaken to verify the presence when doubtful or to get the specimens or the oothecae collected.

A distribution map was plotted and analysed to better understand the dynamics of arrival and spreading (basemap made with Natural Earth data). The Average Nearest Neighbour (ANN) analysis has been done to measure the distance between each mantid presence record centroid and its nearest neighbour's centroid location, calculating all their relative distances. The average distance, related to the average for a hypothetical random distribution, was then calculated to evaluate the degree of clustering of the distribution at different scales. The ANN over the minimum convex polygon between all the known localities in Europe (Italy and France) for *H. patellifera* has been used to test the hypotheses of a random or a definite pattern of distribution for this species for its arrival in Europe. A regular pattern of distribution (dispersed or clustered) would indicate the presence of a specific driving force influencing the arrival of this species over a random and undefined scheme from different and unrelated sources. The minimum convex polygon for the area of Milan, the most densely-populated hot spot for this species in Europe, has been used to measure the randomness of the presence of this species after its arrival in a locality, as a case study. The railway, as a corridor for mantids spreading, has been already hypothesised for other species in this area (Battiston and Buzzetti 2012) and this dynamic has been here investigated. The Individual Range, the mean between the average nearest neighbour distances, calculated with the ANN (3 km) and the minimum directly observed spreading distance/year (1 km), was used to create a buffer of 2 km from the train railways to measure the relative distances from the mantids' localities to the railways or the train depots (data extracted from OpenStreetMap). Each presence record was related to the CORINE Land Cover (CLC) categories (European Environment Agency 2012) to evaluate the actual habitat of this species in Europe. All the mantids' individuals/oothecae, collected or observed in the same locality within a range of 100 m, were considered as a single locality. The appearance of a mantis in or near the same locality for more than a year was considered as a different locality/year. QGIS and ESRI ArcGIS were used to run the GIS analysis.

A comparison of the average temperatures of Milan, Niigata (on the northernmost confirmed distribution limit of this species, from the Niigata Red Data Book (Niigata 2010) and Berlin (the largest railway and commercial centre, close to the northernmost distribution limit of Mantodea in Europe) species: *M. religiosa*, records from: Inaturalist 2019), from the data of NOAA 2019, has been done to evaluate the adaptability of this species to a future spreading in Central and Northern Europe.

The taxonomy of the genus *Hierodula* is far from being definitive, but the morphology of two alien species present in Europe: *H. tenuidentata* and *H. patellifera*, has been here investigated. We examined a total of 16 specimens of *H. patellifera* (11 from Northern Italy, two from Central Italy and three from Jiava, Japan and Thailand for comparison) and eight specimens of *H. tenuidentata* from the Po valley. In addition, a comparison of the male genitalia (as the most discriminative character a priori for Mantodea, see Battiston et al. 2010) of some specimens here collected (Montegrotto Terme) with topotypical specimens from South East Asia (Thailand and Jiava) has been undertaken.

## Results

**Figure 1.**
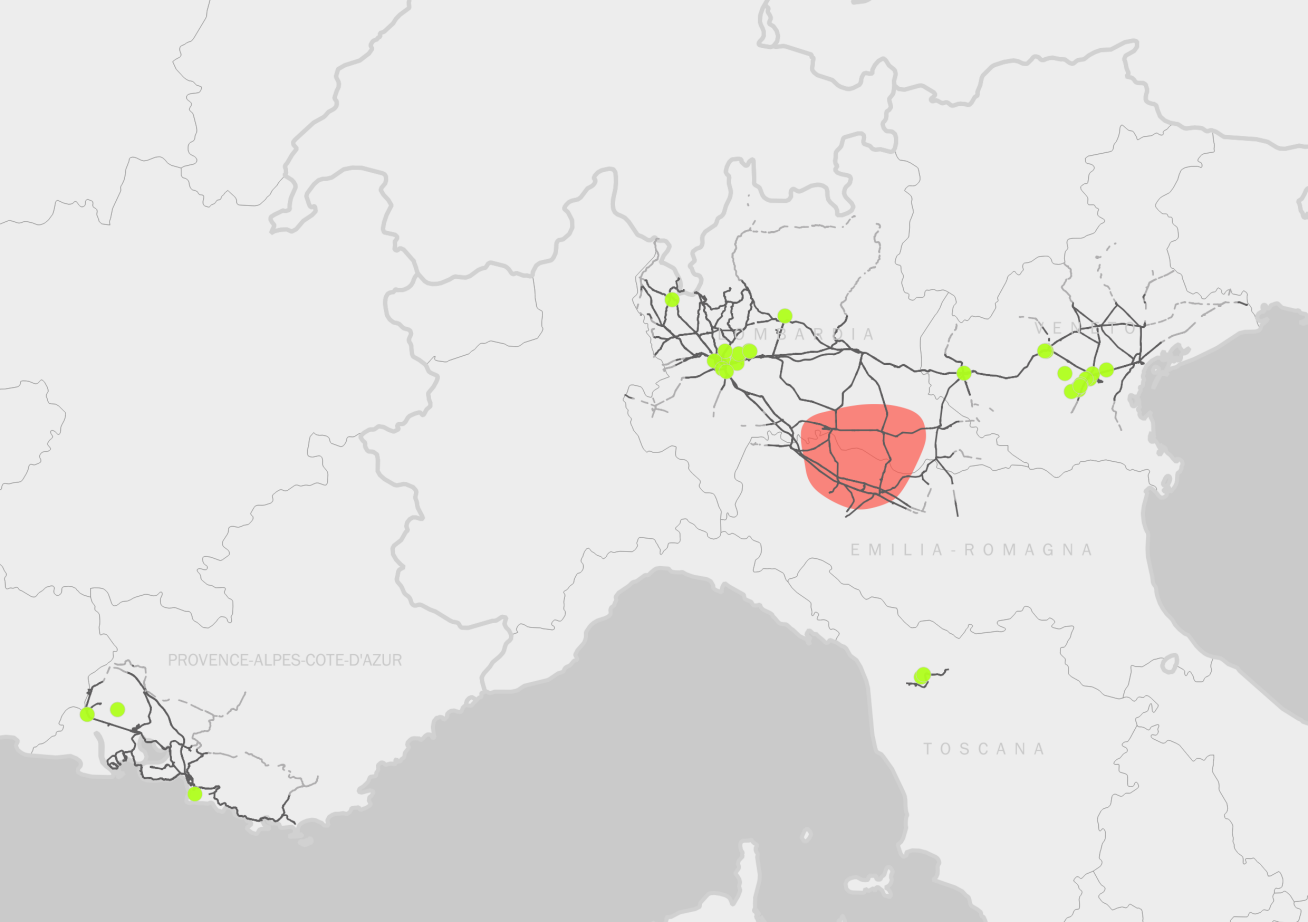
Distribution of *Hierodula* in Europe. *H. patellifera* records (green points) are plotted over the railway network in the occurrence areas (black lines, data from @OpenStreetMap contributors). The red polygon represents the area of distribution of *H. tenuidentata*. Records from France from Moulin 2020. Basemap: Natural Earth data.

**Figure 2.**
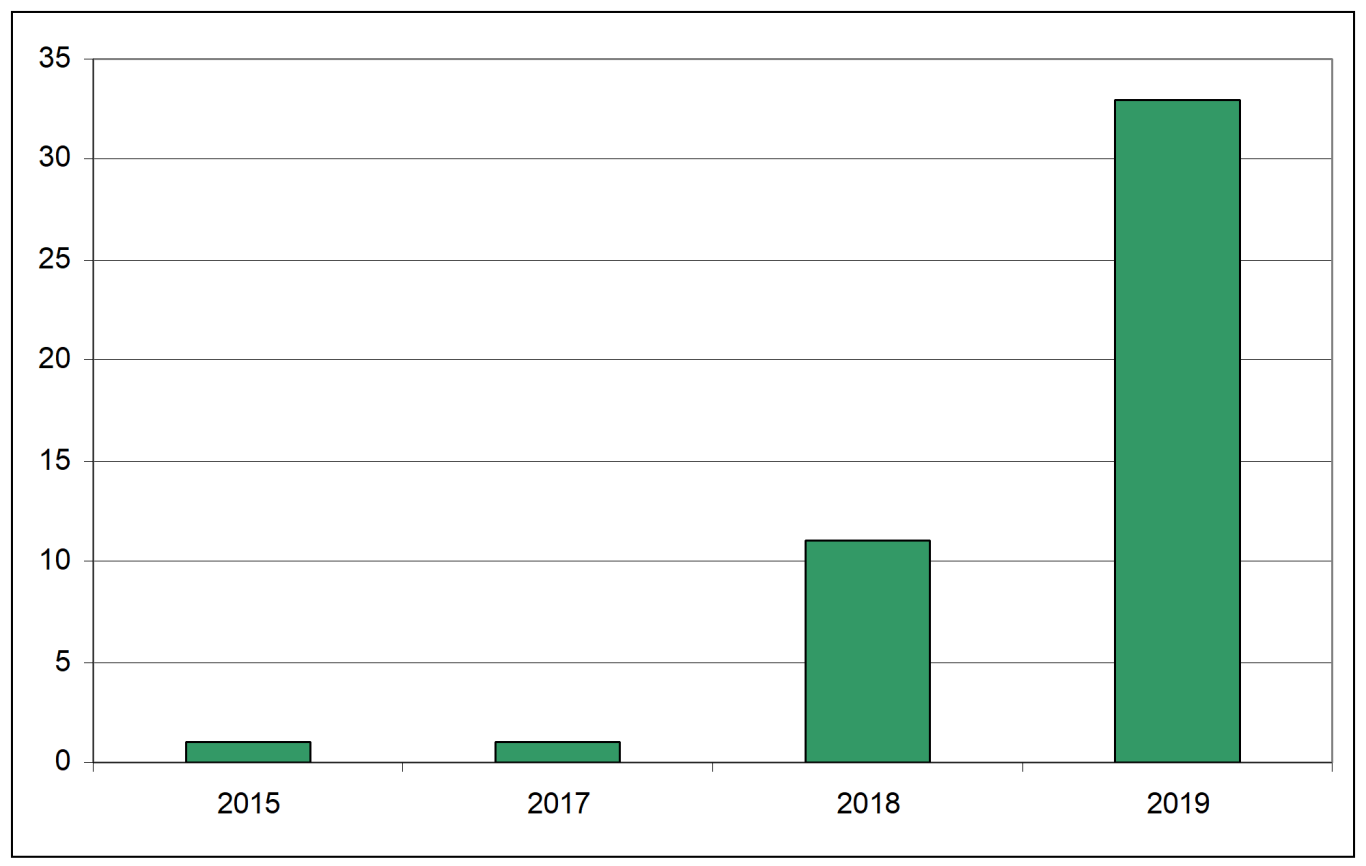
Number of presence records of *H. patellifera* in Italy (y axis) in each year (x axis).

A total of 46 new records of *H. patellifera* has been collected in 39 different localities/years from 2015 to 2019 (Suppl. material 1) and plotted on the map (Fig. 1). The distribution of this species in Europe now covers a total area (minimum convex polygon) of about 90,000 km^2^. In this area, the distribution is clearly not random but clustered in definite and discontinuous localities, as supported by the ANN analysis (Nearest Neighbour Ratio: 0.36, z-score: -7.56, p-value: 0.00). Most of these localities (58.97%) are located less than 2 km from a train railway (84.61% of these along the Milan-Padua railway, one of the main commercial routes in Italy) and about one in three (30.76%) is located less than 2 km from a train depot. However, in the area of Milan (about 125 km^2^), the distribution is random, with a tendency to dispersion (Nearest Neighbour Ratio: 1.89; z-score: 5.93; p-value: 0.00). The species was first observed in France in 2013 with a single female individual (Moulin 2020) and a single individual appeared during September 2015 again in France, but also in a suburb of Milan (Gorgonzola). Then the frequency of encounters dramatically increased in the following years in Northern Italy (Fig. 2).

Both juveniles and adults of *H. patellifera* were found on trees in urban environments (e.g. backyards, along tree-lined streets, urban riverbanks) or adjacent areas (e.g. in a mulberry cultivation for sericulture in Padua or in olive groves in France). Specimens were sighted and collected during the day or just after sunset, wandering on the roadsides (three individuals were found roadkilled) or on vegetation (resting on the abaxial side of leaves or on the branches or climbing up the tree trunks).

The main discriminative character to separate *H. patellifera* from *H. tenuidentata* is confirmed to be the fore coxa morphology with three to four yellow-whitish callosities on the base of the delicate coxal spines in *H. patellifera*, absent under the stronger spines of *H. tenuidentata* (Table 1). In addition, the two species differ in male genitalia: in the process of the right phallomere (R1a), more triangular in *H. tenuidentata*, in the shape of the pseudophallus (afa), clearly shorter and more rounded in *H. tenuidentata* and in the posterior process of the ventral phallomere (pda), longer and more tapered in *H. patellifera* (Fig. 3). The morphology of the Italian population is well compatible with the specimens of *H. patellifera* from South East Asia.

## Discussion

**Figure 4.**
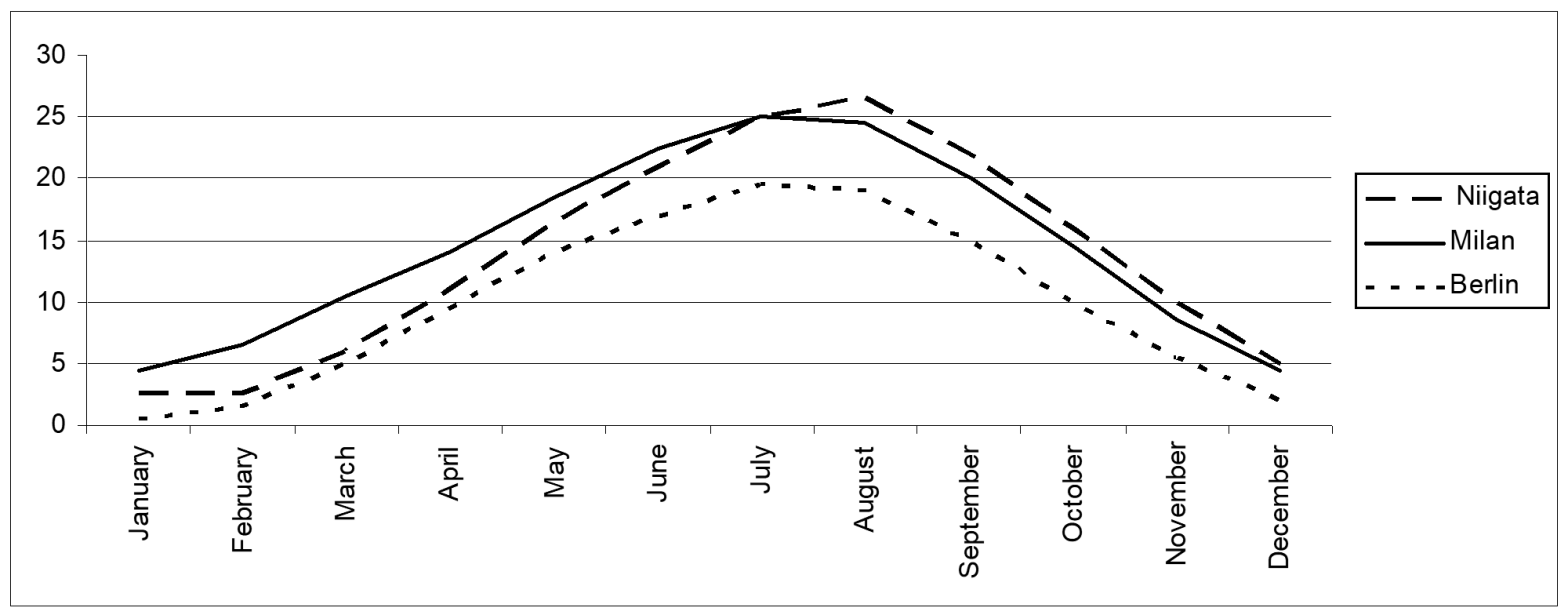
Average temperatures (°C) recorded during 2019 in the northernmost distribution of *H. patellifera*: in Japan (Niigata Prefecture), in Italy (Milan) and in central Europe (Berlin). Data fromNOAA 2019.

In Northern Italy, as in many other European areas, the 2018-2019 winter has been problematic for some insects groups. A climate anomaly of warm weeks alternated by very cold ones, reaching the late spring of 2019, has decimated, for instance, many bee populations, almost erasing all the spring honey production for Northern Italy (Pappalardo and Naldi 2019). Oothecae of *H. patellifera* seem, however, to have well survived this challenging season. This species has been present in France since 2013, but the climate of Provence is mostly mild, Mediterranean. This species, in its natural habitat, reaches localities (e.g. in Northern Japan in the prefecture of Niigata) with a climate comparable to the continental one of Northern Italy and Central Europe (Fig. 4), where an expansion is expected to occur in the next years.

**Figure 5.**
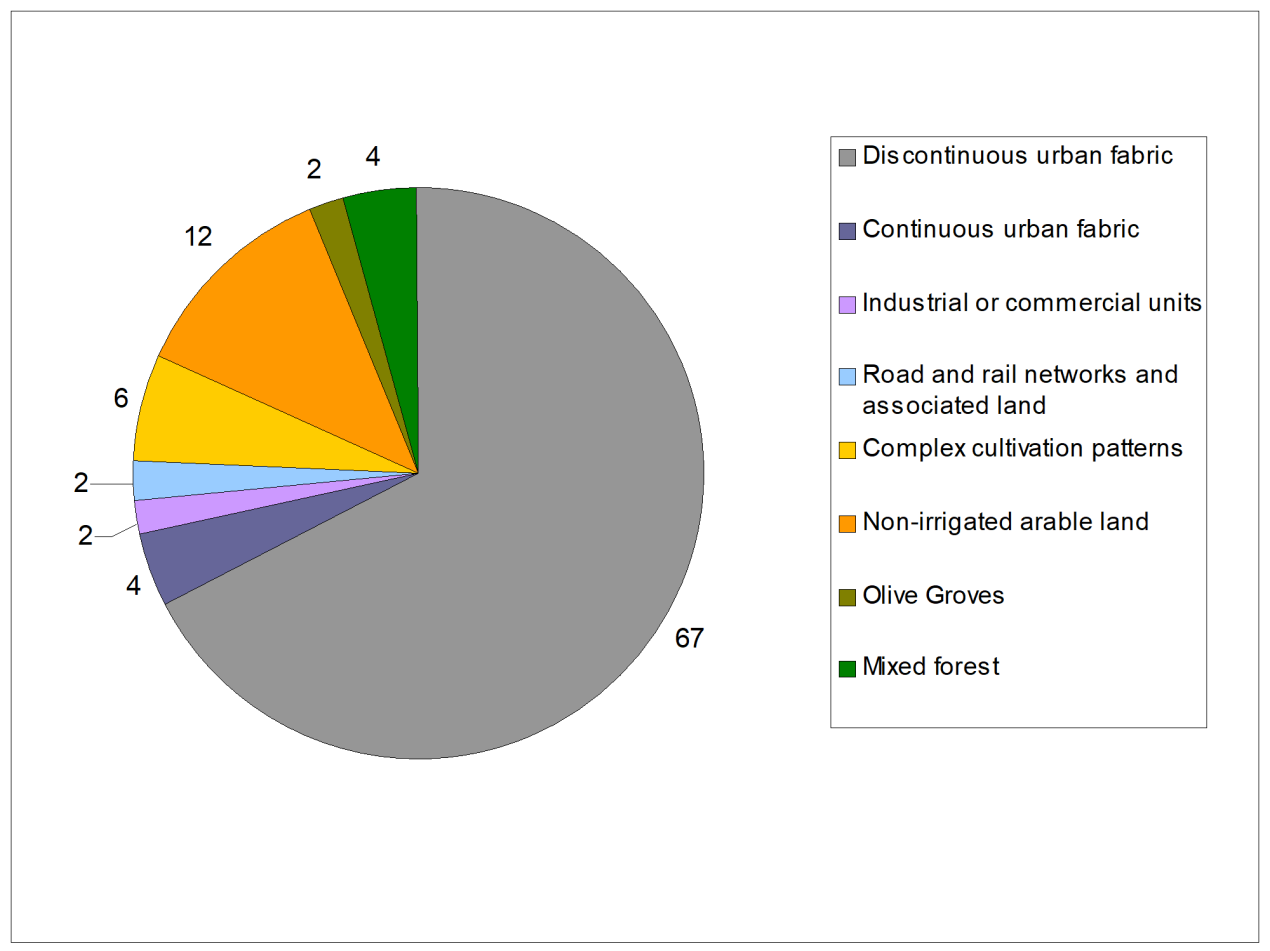
Habitat in Europe of *H. patellifera*, from the presence records over the CORINE Landcover categories. Values are in percent.

In Italy, the species has been present at least since 2015, from the oldest records here collected. Its ability to survive in a more extreme climate and spread in anthropised environments (76% of the presence records are distributed on the “artificial surfaces” class of the CLC nomenclature, including the 67% of them on discontinuous urban fabric, Fig. 5), something they also share with *H. tenuidentata* (Battiston et al. 2018), proves that its presence should now be considered definitely stable in Europe and increasing in many different and localised populations. Gardens of residential areas in a discontinuous urban fabric seem to be, at present, the most-used habitat of this species in Europe.

There are still not enough elements to define the real origin of this already complex situation but there are some clues. Adult individuals of this species have been recorded resting on containers in France (Moulin 2020), on local passenger trains in Italy and some oothecae, compatible with this species, have been collected on bamboo brooms shipped to Italy from Asia, where this species naturally occurs. From the spatial analyses, this multivariate dynamic seems to be confirmed. The species seems to have arrived on Asian goods in single defined localities using, probably, a commercial route which may be cargo ships at first, then the railway network. Traditional roads for cars and trucks should also be considered, but the proximity to train depots of many of the records suggests a preference for the rail transport system. It must be noted, however, that this dynamic may not be the exclusive explanation for this distribution. Few but remarkable occurrences have been recorded far from train depots, railways or even towns. In some other cases, the records were very close to a train depot or railway on the map, but separated from it by evident barriers (i.e. a large river). Most of these were females or both males and females, but never males alone, good fliers which would have overpassed these barriers. Together with passive transport by humans, railways and roadside verges should also be considered for active spreading, if not in this early phase, maybe in the future. These alternative ecological corridors have been recently suggested to help the spreading of some insects, including mantids like *Ameles spallanzania* (Rossi 1792) and *M. religiosa* (Battiston and Buzzetti 2012, Bolshakov et al. 2010)

If spatial data are considered carefully ona large scale, once arrived in a city, the presence of this species is clearly randomly dispersed, probably following not a natural expansion from a single hot spot of arrival (like a train depot or a commercial warehouse), but more likely again human mediated, following the micro-commerce of some goods, maybe bamboo made artefacts or exotic plants, sold randomly to local stores, then to the final consumers, their houses or gardens in residential areas (discontinuous urban fabric).

**Figure 6.**
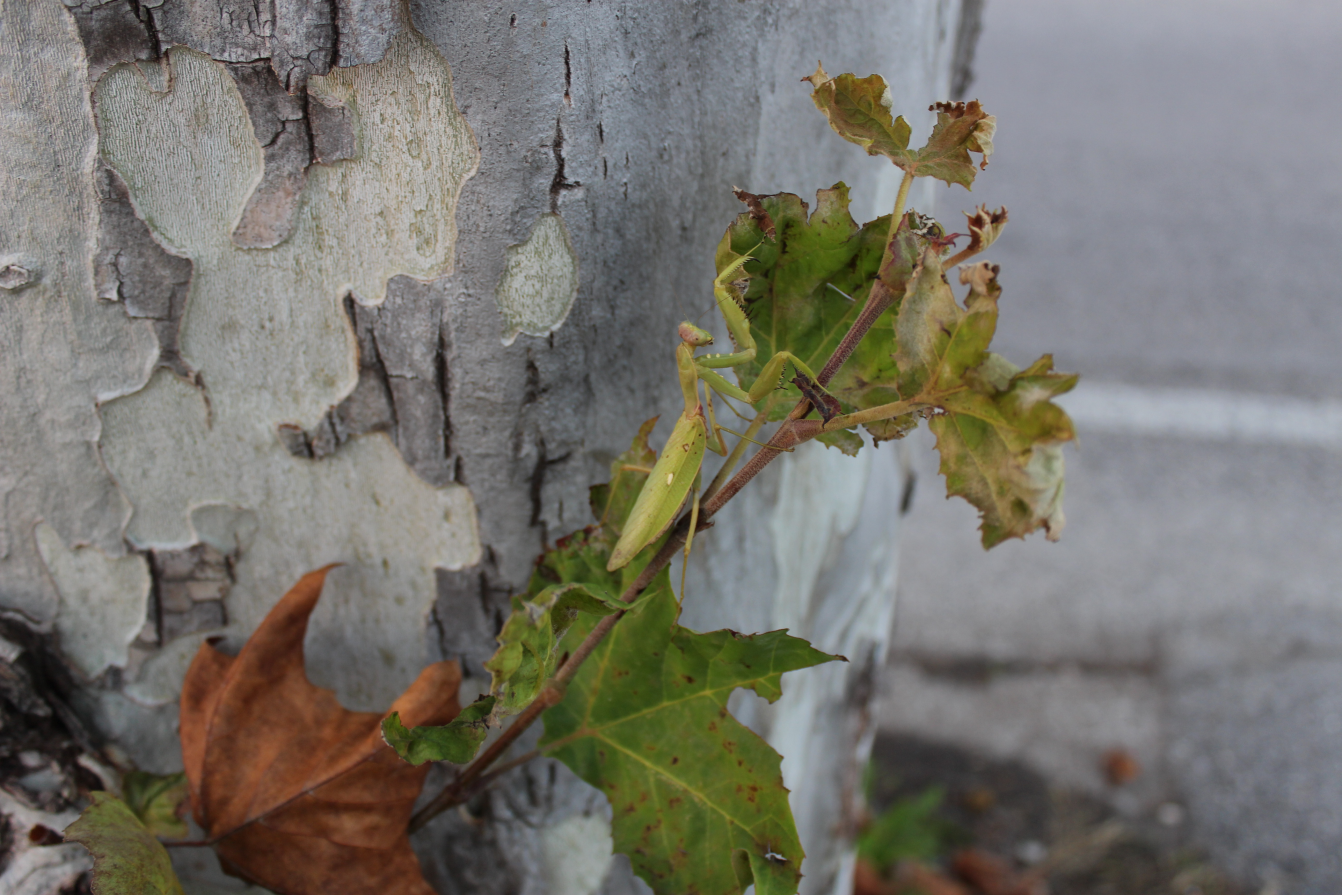
A female of *H. patellifera* climbing an American sycamore (*Platanus occidentalis* L., 1753) on a roadside in the city of Vicenza (Italy).

The impact of this species on the European ecosystems is far from being understood. Some concerns about the competition over the local mantids have been already raised (Battiston et al. 2018, Moulin 2020, Schwarz and Ehrmann 2018). Nonetheless, this species already shares some localities with *M. religiosa* in Asia (Ehrmann 2002, Niigata 2010) but the more arboreal habits of *H. patellifera* (Fig. 6), something often observed also in Europe, may keep them separate, as occurs in Asia. It must be noted, however, that both species have been recorded here in the urban fabric, sometimes a few metres from one another and with an almost identical life cycle. If, in nature, *Mantis* and *Hierodula* fit their separate ecological niches, in an artificial one, the games may be still open and further studies should be done on this.

## Supplementary Material

4D024762-F846-5A89-B9FA-4140F2ACDEAF 10.3897/BDJ.8.e50779.suppl1 Supplementary material 1 *Hierodula patellifera* European records Data type: occurrences
File: oo_377303.csv https://binary.pensoft.net/file/377303 Battiston Roberto, Amerini Rachele, Di Pietro William, Guariento Luis Alessandro, Luca Bolognin, Moretto Enzo

## Figures and Tables

**Figure 3a.**
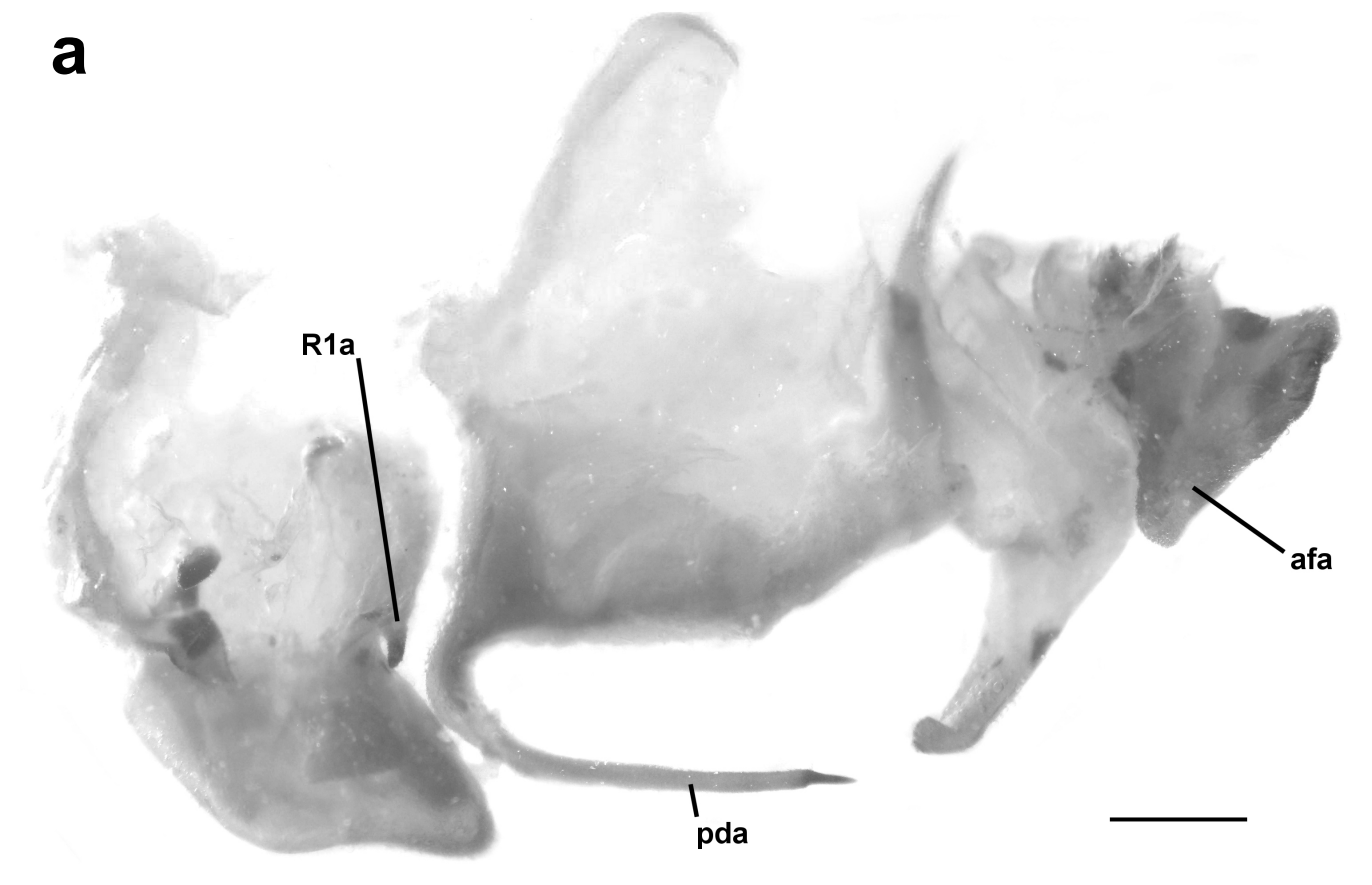
*Hierodula patellifera*

**Figure 3b.**
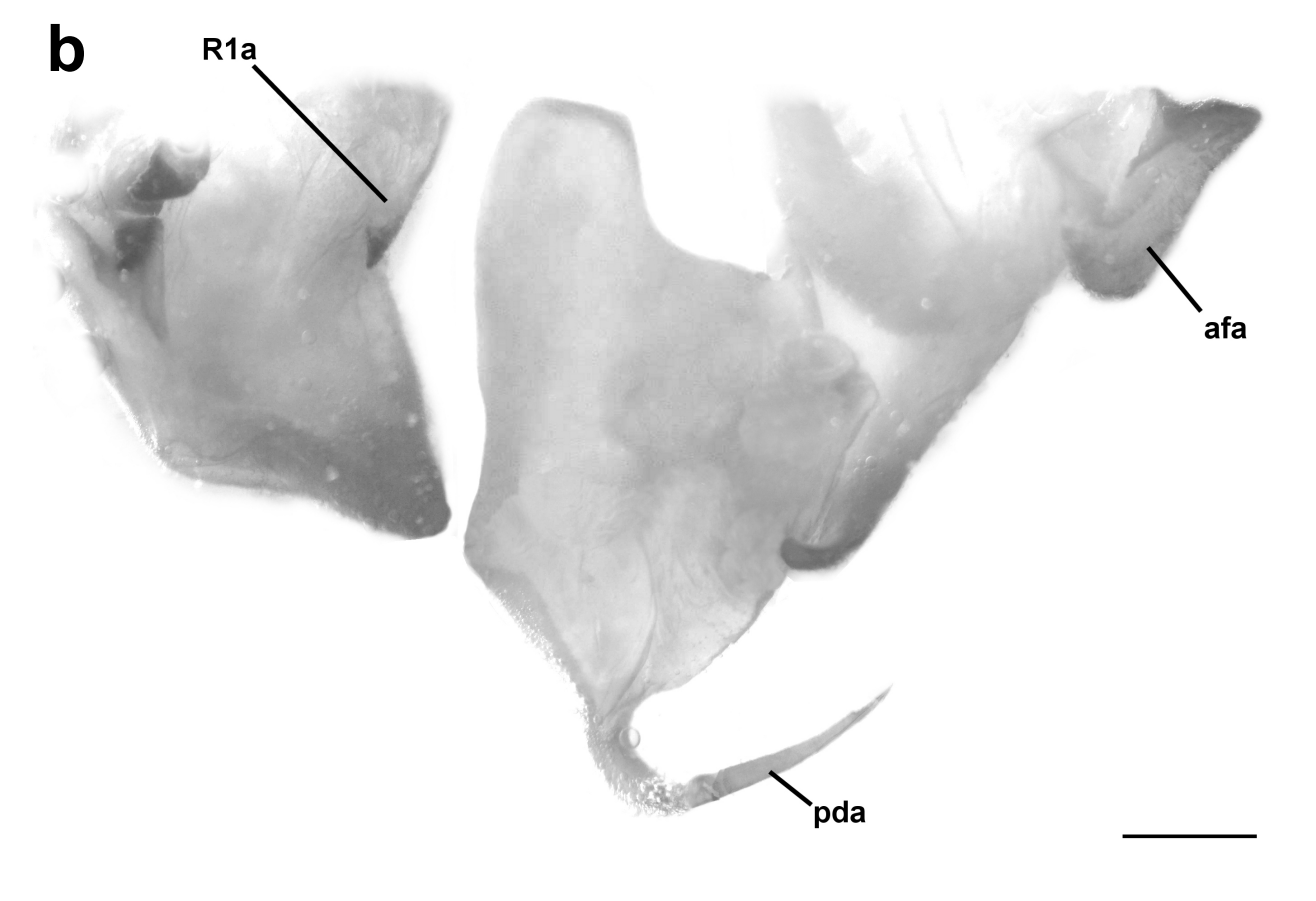
*Hierodula tenuidentata*

**Table 1.** Key to the species of the mantids in the subfamily Mantinae present in Europe, native and introduced with confirmed and stable populations.

1	Body slender. Pronotum very long, more than 5 times longer than large. Frontal sclerite at least two times broader than high. Wings opaque.	*Tenodera sinensis*
-	Body less slender. Pronotum shorter, less than 4 times longer than large. Frontal sclerite less than 2 times broader than high. Wings hyaline.	2
2	Presence of an evident dark or dark-ringed spot, on the inner side of the front coxae, clearly visible even in the last juvenile stages. Pronotum slender and stigma of the same colour as the tegminae.	*Mantis religiosa*
-	Absence of an evident dark spot on the front coxes. Pronotum short with curved margins. Stigma on the tegminae whitish.	3
3	Pronotum with evident narrowing before the well-marked supracoxal dilation.	*Sphodromantis viridis*
-	Pronotum short with expanded margins and sub-ovoid profile without an evident narrowing before the supracoxal dilation	4
4	Inner margin of coxes with delicate spines, 3 or 4 of which with an evident yellowish plate at the base.	*Hierodula patellifera*
-	Inner margin of coxes with strong spines but without basal plates.	*Hierodula tenuidentata*
